# Mood instability and mental health service use in autism and attention-deficit/hyperactivity disorder: a natural language processing analysis of CRIS electronic healthcare records from 21 906 children and adolescents

**DOI:** 10.1136/bmjment-2026-302488

**Published:** 2026-06-18

**Authors:** Asilay Seker, Seungyoung Kim, Susie Chandler, Craig Colling, Rashmi Patel, Edmund Sonuga-Barke, Johnny Downs

**Affiliations:** 1 CAMHS Digital Lab, King’s Maudsley Partnership, King’s College London, London, UK; 2 Department of Child and Adolescent Psychiatry, Institute of Psychiatry, Psychology & Neuroscience, King's College London, London, UK; 3 Institute of Psychiatry, Psychology and Neuroscience, King’s College London, London, UK; 4 South London and Maudsley NHS Foundation Trust, London, UK; 5 Department of Psychiatry, University of Cambridge, Cambridge, UK; 6 National Institute for Health Research (NIHR) Biomedical Research Centre, South London and Maudsley NHS Foundation Trust, London, UK

**Keywords:** Attention Deficit and Disruptive Behavior Disorders, Child Development Disorders, Pervasive, Mental Health Services, Neurodevelopmental Disorders

## Abstract

**Background:**

Children and young people (CYP) with neurodevelopmental diagnoses such as autism spectrum disorder (ASD) and attention-deficit/hyperactivity disorder (ADHD) have high child and adolescent mental health service (CAMHS) needs. Mood instability is a common and impairing emotion dysregulation-related symptom linked to increased adult psychiatric service use; however, its role in CAMHS trajectories remains unclear. We aimed to examine whether baseline mood instability was significantly associated with time to discharge and annual CAMHS use in CYP with ASD and/or ADHD.

**Methods:**

We applied natural language processing (NLP) to extract mentions of mood instability within 3 months of ASD or ADHD index diagnosis from electronic health records of 21 906 CYP referred to CAMHS between 2008 and 2022. We used accelerated failure time models and negative binomial regression to assess associations between baseline mood instability and time to discharge and annual CAMHS use, adjusting for clinical and sociodemographic confounders.

**Findings:**

Mood instability was associated with increased annual CAMHS use across ASD (adjusted incidence rate ratio (aIRR) 1.24, 95% CI 1.08 to 1.42), ADHD (aIRR 1.47, 95% CI 1.30 to 1.67) and ASD+ADHD (aIRR 1.27, 95% CI 1.12 to 1.44) groups. While mood instability had no significant effect on discharge timelines in autistic children with or without ADHD, it was linked to reduced time to discharge in the ADHD group (aTR 0.76, 95% CI 0.69 to 0.84). Associations were most pronounced in those not receiving ADHD medication in the ADHD group (aIRR 1.67, 95% CI 1.47 to 1.89; aTR 0.70, 95% CI 0.61 to 0.79).

**Conclusions:**

Mood instability was significantly associated with elevated CAMHS use in CYP with neurodevelopmental conditions, with differential effect across diagnostic groups. This may reflect both variations in clinical expression of mood instability and configuration of neurodevelopmental CAMHS provision.

**Clinicalimplications:**

These findings suggest the importance of assessing emotion dysregulation in care planning and pathway allocation in neurodevelopmental CAMHS. NLP offers a time- and cost-efficient approach to surface and structure clinical data from electronic CAMHS records for scalable clinical research on complex constructs such as mood instability.

**WHAT IS ALREADY KNOWN ON THIS TOPIC**

**WHAT THIS STUDY ADDS**

- NLP-identified mood instability at the time of diagnosis was associated with higher annual CAMHS use in CYP with autism and/or ADHD.
- This effect was most pronounced in the ADHD-only group, where mood instability was also linked to a shorter time to discharge; these associations were strongest in CYP not receiving ADHD medication.

**HOW THIS STUDY MIGHT AFFECT RESEARCH, PRACTICE OR POLICY**

- Clinicians should be aware of the associations between baseline mood instability in CYP and more frequent CAMHS use, which may be attenuated in those receiving interventions for underlying difficulties, such as medication for ADHD.
- Care pathway allocation for CYP with ADHD-only requires further consideration in the context of baseline mood instability, particularly if they are not receiving ADHD pharmacotherapy.
- NLP methods show great potential for analysing emotion dysregulation-related constructs in CYP at scale for clinical research.

## Introduction

 Attention-deficit/hyperactivity disorder (ADHD), a neurodevelopmental condition characterised by hyperactivity, attention difficulties and impulsivity, affects up to 7% of children and young people (CYP).^1^ Autism (referred to as autism spectrum disorder (ASD) in line with the Diagnostic and Statistical Manual of Mental Disorders, Fifth Edition and the diagnostic practice in child and adolescent mental health services (CAMHS)) is another prevalent neurodevelopmental condition, with reported rates as high as 1.76% in England, presenting with social-communication differences, restricted and repetitive interests and behaviours, and sensory sensitivities.^2 3^ Those with ASD and/or ADHD are more likely to use mental health services than their typically developing peers, for core difficulties and frequently co-occurring challenges like emotion dysregulation, self-harm, depression or anxiety.^4 5^ ADHD and/or ASD may be present in as many as 25% of CYP under English CAMHS care,^6 7^ a figure expected to rise with improved recognition and increasing administrative prevalence.^8 9^ However, as highlighted in the 2024 Children’s Commissioner Report, CAMHS are struggling to meet the growing mental health needs of neurodivergent CYP, from assessment through to postdiagnostic support.^10 11^ This demand further strains CAMHS capacity and has a knock-on effect on waitlists for new referrals.^12^,^14^ In the UK, for example, the average wait time for neurodevelopmental assessments has surpassed 500 days.^15^ Given these challenges, understanding what drives CAMHS use in this group is crucial for improving service delivery and clinical outcomes. However, research into transdiagnostic factors influencing CAMHS needs across neurodevelopmental conditions remains scarce.

One such underexplored factor in this context is emotion dysregulation, a highly common and impairing difficulty and a significant risk factor for adverse mental health outcomes among CYP with ASD or ADHD.^16 17^ Emotion dysregulation may contribute to elevated CAMHS utilisation in these populations by mediating the development of co-occurring mental health conditions or by directly impacting the frequency and duration of service contacts.^18^,^20^ Within the broad construct of emotion dysregulation, mood instability stands out as a common and clinically salient presentation.^21 22^ Mood instability is described as marked mood reactivity in the Diagnostic and Statistical Manual, Fourth Edition—it mainly refers to dysregulated mood with extreme and/or frequent fluctuations over time,^23^ and has indeed been associated with mental ill-health and increased psychiatric service use in adolescents and young adults.^2224^,^26^ Mood instability was suggested to have trait-like properties in neurodevelopmental conditions such as ADHD, and may benefit from pharmacotherapy as core ADHD symptoms.^27^,^30^ Emotion dysregulation is among core clinical concerns in autistic young people too, with established links to co-occurring psychiatric presentations.^31^,^33^ While research on mood instability in autism is scarce, severe mood problems, including labile mood, have been linked to psychopathology in ASD.^34^ In brief, despite its likely relevance, the specific role of mood instability in shaping mental healthcare needs of CYP with neurodevelopmental conditions remains significantly underexplored.

An important barrier to studying emotion dysregulation, or more specifically mood instability, in clinical samples at scale is their lack of structured assessment in CAMHS.^28^ While highly common in CYP with neurodevelopmental conditions, these symptoms are not formally recognised within standard neurodevelopmental assessments.^35 36^ Mood instability, although commonly flagged in clinical notes when present, is rarely measured using standardised instruments in CAMHS.^22^ As a result, the availability of many questionnaire-based emotion dysregulation measures does not translate into routine clinical monitoring, and these symptoms often still need to be inferred from clinician notes for service evaluation or research purposes. While CAMHS records typically contain rich clinical data, manual review of these is not feasible in routine practice. Innovative approaches such as natural language processing (NLP) offer a practical solution to automate electronic health record (EHR) analyses.^37^ As previously shown in adult and adolescent samples, NLP can structure EHR notes into analysable data to retrospectively screen complex constructs including mood instability.^22 26^ Considering 80% of clinical information is stored in free text, particularly in paediatric healthcare, NLP-extracted information presents a valuable resource for clinical researchers.^38^ However, despite their potential and more frequent examples of use in paediatric studies, NLP methods are rarely applied in CAMH research.

To address these gaps, we explored the utility of NLP to identify mood instability in CAMHS records. We then examined overall and diagnosis-specific effects of baseline mood instability on two key community CAMHS metrics in CYP with ASD and/or ADHD, reflecting duration and annual rate of service use.

## Methods

### Study design, sample and setting

We conducted a retrospective cohort study on the Clinical Record Interactive Search (CRIS) of EHRs from the South London and Maudsley NHS Foundation Trust (SLaM). CRIS holds de-identified data of SLaM CAMHS patients from 2007 onwards and is governed by an oversight committee.^39^,^41^ The sample was CYP referred to SLaM CAMHS between 1 January 2008 and 31 December 2022.

SLaM CAMHS are organised into 10 main pathways (online supplemental file 1), with ASD and ADHD managed under two separate pathways. Although certain care practices and pathway labels may have evolved over the study period, the core configuration of services for these two conditions is not considered to have changed substantially. Both pathways offer specialist diagnostic assessment followed by psychoeducation. Multidisciplinary provision is more typically embedded in ASD teams, where specialist or ASD-informed support is described for CYP with additional ASD-related mental health presentations. In the ADHD pathway, the main intervention remains pharmacotherapy, and any further or alternative input required is likely to warrant an internal or external referral to the general practitioner or another relevant CAMHS pathway, rather than being managed within the same ADHD team.

### Participants

Participants comprised CYP referred to SLaM CAMHS and received a diagnosis of ASD (F84.xx) or ADHD (hyperkinetic disorders) (F90.xx) according to the International Statistical Classification of Diseases and Related Health Problems, 10th edition (ICD-10) in 2008–2022 before their 18th birthday (index diagnosis).^42^ As is established practice in CRIS studies, diagnostic data derived from ICD codes in structured diagnostic fields were supplemented with outputs from the Diagnosis NLP application. Previous CRIS CAMHS research reported precision values of 0.86 for ASD and 0.82 for ADHD.^43^,^45^ 58% of ASD and 48% of ADHD diagnoses in the cohort are available from ICD codes, which is consistent with other ongoing CRIS work. As SLaM CAMHS typically serves CYP between age 5 and 18 years, children diagnosed before the age of 5 years were excluded from the final analysis. Individuals who were admitted to a SLaM inpatient unit within the study window were also excluded, resulting in a final analytical sample of 21 906 participants (online supplemental Figure S1).

### Measures

#### Primary outcomes

Main service use outcomes of interest were the time to discharge from CAMHS and postdiagnostic frequency of annual community CAMHS contacts, both of which were obtained from structured CRIS fields:

- Time to discharge: calculated in years from index diagnosis date to discharge based on discharge dates extracted from CRIS.
- Frequency of annual CAMHS contacts (annual CAMHS use): the total number of face-to-face contacts with CAMHS from the index diagnosis date until discharge or window end date (whichever was earlier), offset against the time under CAMHS. Time under CAMHS was calculated by subtracting index diagnosis date from either discharge date or window end date (whichever was earlier).

### Main exposure

#### Mood instability

Mood instability was identified from CAMHS EHRs via a previously developed NLP approach. This TextHunter-based NLP algorithm identified mentions of mood instability-related terms documented in free-text EHRs (clinical notes, correspondence) within 3 months before/after index diagnosis and provided a binary output for mood instability as present/absent for each participant (Online Supplemental Material). Details of the development and performance metrics of this tool were described in previous work including young people, indicating a precision of 0.91 and recall of 0.72.^26^ This corresponds to an F1-score (harmonised mean of precision and recall) >0.80, which indicates robust performance close to human agreement on the same task.^46^

### Clinical and sociodemographic covariates

Potential confounders of the associations between mood instability and outcomes of interest were decided based on clinical judgement, existing literature and prior CRIS work that examined SLaM CAMHS data.^6 22^ These included gender, age, ethnicity, socioeconomic status, co-occurring psychiatric diagnoses and psychotropic medication use.

Sociodemographic data such as gender, age at index date, ethnicity and socioeconomic status, and clinical variables were extracted from structured fields within CRIS. Clinical variable data were substituted with NLP data surfacing methods where possible to optimise data availability. Index date was defined as the earlier of ADHD or ASD diagnosis date. Age at index diagnosis was calculated by subtracting the date of birth from the index date. Ethnicity was recorded based on UK Office for National Statistics (ONS) categories.^47^ Nine ethnicity categories were collapsed into five to improve statistical sensitivity and participants with ethnicity ‘not stated’ were also included as a separate category, consistent with ONS categorisation of ethnicity and prior research using CRIS.^2248^,^50^ Socioeconomic status was derived from neighbourhood deprivation (based on the patient’s residential postcode), which was measured using multiple indices of deprivation for small areas.^51^ These indices combine weighted indicators of income, employment, education, health, crime, barriers to housing and services and living environment into a single deprivation score.

The presence or absence of co-occurring multiaxial ICD-10 psychiatric diagnoses were extracted from structured data fields and recorded as binary variables. These included psychosis, depression, eating disorder, obsessive-compulsive disorder (OCD), phobia, anxiety, intellectual disability, conduct disorder and emotional disorders (Online Supplemental Material).^42^ ADHD, ASD and psychosis diagnoses were also supplemented by NLP outputs where available.^52^ Pharmacological treatment provision including antipsychotic, antidepressant, hypnotic and ADHD medication within 12 months of diagnosis was extracted from free-text using NLP tools into a binary variable.^52^

### Statistical analysis

The data were analysed using Stata (V.18.0). As missing data were well below 5% for all variables included in the analysis (online supplemental Table S1), we conducted a complete case analysis.^53^ Descriptive statistics were reported as means and medians for continuous variables depending on distribution and as frequencies and percentages for categorical variables. Diagnostic groups were compared with analysis of variance or Kruskal-Wallis tests for continuous variables, as appropriate, or χ^2^ tests for categorical variables. Following interaction analyses between mood instability and diagnostic group for each outcome (online supplemental Table S2 and S3), the data were stratified into three diagnostic groups of ASD-only (ASD), ADHD-only (ADHD) and co-occurring ASD and ADHD diagnoses (ASD+ADHD) groups for subsequent analyses.

For time-to-discharge outcome, we conducted an accelerated failure time (AFT) model-based time-to-event analysis. AFT models directly relate the main exposure and covariates to survival time and yield time ratios (TRs), where TR <1 indicates a shorter and TR >1 a longer time to discharge compared with the reference group (eg, TR 0.90 corresponds to a 10% shorter time to discharge). We chose AFT models rather than Cox proportional hazards models as our primary interest was in quantifying how long CYP remained in CAMHS and discharge occurred in the majority of the cohort, making TRs more clinically relevant than HRs. Following comparisons between distributions based on Akaike Information Criterion and Bayesian Information Criterion model fit indices, where lower values indicate better model fit (online supplemental Table S4), time ratios to discharge were examined via univariate and multivariable Weibull AFT models.

Negative binomial regression was used to analyse annual CAMHS use, accounting for overdispersion in the data. For this, the number of postdiagnostic CAMHS contacts was used as the dependent variable offset against years spent under CAMHS, with sociodemographic and clinical covariates fitted in the models where appropriate.

### Sensitivity analyses

Sensitivity analyses were conducted by stratifying the ADHD and ASD+ADHD groups based on ADHD medication status to assess whether the association between mood instability and outcomes differed by medication use. These models included the same covariates as the main analyses.

## Results

### Descriptive characteristics

Table 1 shows the descriptive characteristics of the study sample across the ASD, ADHD and ASD+ADHD groups, missing values are represented in online supplemental Table S1. The sample was predominantly male and of white ethnic background. The majority of autistic CYP came from more affluent neighbourhoods, while the ADHD group mostly resided in more deprived areas. The most common co-occurring diagnosis was intellectual disability in autistic groups, whereas emotional disorders were most common in the ADHD group. The most frequently prescribed medications were ADHD medications in CYP with ADHD, and antidepressants in the ASD group.

**Table 1:** Sociodemographic and clinical characteristics of study sample

	Total n (%) 21 906(100.0%)	ASD n (%) 7674(35.0%)	**ADHD n (%)** **7889**(36.0%)	**ASD+ADHD n (%)** **6343**(29.0%)	P value
**Age at index date, mean (SD**)	11.96(0.02)	12.48(0.04)	11.89(0.04)	11.43(0.04)	<0.001
**Gender**					<0.001
Male	14 209(64.9%)	4577(59.7%)	5333(67.6%)	4299(67.9%)	
Female	7596(34.7%)	3050(39.8%)	2535(32.2%)	2011(31.7%)	
Other	77(0.4%)	36(0.5%)	16(0.2%)	25(0.4%)	
**Ethnicity**					<0.001
White	10 438 (47.7%)	3671(47.9%)	3449(43.8%)	3318(52.4%)	
Black	4596(21.0%)	1610(21.0%)	1752(22.3%)	1234(19.5%)	
Asian	932(4.3%)	411(5.4%)	252(3.2%)	269(4.2%)	
Mixed	2469(11.3%)	746(9.7%)	961(12.2%)	762(12.0%)	
Not stated	2604(11.9%)	1008(13.1%)	1164(14.8%)	432(6.8%)	
Other	840(3.8%)	222(2.9%)	296(3.8%)	322(5.1%)	
**Neighbourhood deprivation**					<0.001
First (least deprived)	5236(24.6%)	2058(27.6%)	1593(20.7%)	1585(25.8%)	
Second	5358(25.1%)	1879(25.2%)	1941(25.2%)	1538(25.0%)	
Third	5385(25.3%)	1808(24.2%)	2044(26.5%)	1533(24.9%)	
Fourth (most deprived)	5331(25.0%)	1715(23.0%)	2121(27.5%)	1495(24.3%)	
Mood instability	3408(15.6%)	1042(13.6%)	1089(13.8%)	1277(20.1%)	<0.001
Time to discharge (years), median (IQR)	0.74 (0.27–1.66)	0.62 (0.22–1.35)	0.82 (0.30–1.92)	0.81 (0.31–1.86)	<0.001
Total CAMHS use, median (IQR)	3 (0–12)	2 (0–10)	3 (0–11)	4 (1–14)	<0.001
Annual CAMHS use, median (IQR)	3.68 (0–11.68)	4.07 (0–13.71)	3.52 (0–10.29)	3.58 (0.72–10.81)	<0.05
Co-occurring diagnoses					
Eating disorder	515(2.4%)	239(3.1%)	159(2.0%)	117(1.8%)	<0.001
Psychosis	932(4.3%)	324(4.2%)	294(3.7%)	314(5.0%)	0.002
OCD	698(3.2%)	398(5.2%)	128(1.6%)	172(2.7%)	<0.001
Affective disorder	1147(5.2%)	472(6.2%)	376(4.8%)	299(4.7%)	<0.001
Phobia	253(1.2%)	112(1.5%)	57(0.7%)	84(1.3%)	<0.001
Anxiety	1692(7.7%)	739(9.6%)	429(5.4%)	524(8.3%)	<0.001
Emotional disorder	1707(7.8%)	525(6.8%)	709(9.0%)	473(7.5%)	<0.001
Intellectual disability	1908(8.7%)	876(11.4%)	336(4.3%)	696(11.0%)	<0.001
Conduct disorder	1272(5.8%)	212(2.8%)	566(7.2%)	494(7.8%)	<0.001
Tic disorder	246(1.1%)	64(0.8%)	82(1.0%)	100(1.6%)	<0.001
**Medication use**					
ADHD medication	4841(22.1%)	0(0.0%)	2647(33.6%)	2194(34.6%)	<0.001
Antipsychotic medication	1076(4.9%)	469(6.1%)	179(2.3%)	428(6.7%)	<0.001
Antidepressant use	1469(6.7%)	768(10.0%)	275(3.5%)	426(6.7%)	<0.001
Hypnotic use	1148(5.2%)	372(4.8%)	288(3.7%)	488(7.7%)	<0.001

Missing values are reported for each diagnostic group in online supplemental table S1. P values are from one-way analysis of variance for continuous variables and Pearson’s χ2 tests for categorical variables.

ADHD, attention-deficit/hyperactivity disorder; ASD, autism spectrum disorder; CAMHS, child and adolescent mental health services; OCD, obsessive-compulsive disorder.

Mood instability rates were similar in ASD (13.6%) and ADHD (13.8%) groups, but greater in the ASD+ADHD group (20.1%). Median total CAMHS use (unadjusted for follow-up time) was highest in the ASD+ADHD group, followed by ADHD and then ASD. Median time to discharge was longest in the ADHD group and shortest in the ASD group.

### Time to discharge from CAMHS

Kaplan-Meier curves presented in online supplemental Figure S2 display the probability of remaining under CAMHS over the observation period, stratified by baseline NLP-identified mood instability status. These curves suggest that CYP with mood instability leave CAMHS sooner than those without (log-rank p<0.001) in the total sample. However, in subsequent analyses stratified by diagnostic group, only in the ADHD group was baseline mood instability associated with significantly earlier discharge (log-rank p<0.001).

Weibull AFT analyses showed that in the entire sample, mood instability was again associated with a significantly reduced time to discharge in both the unadjusted (TR 0.87, 95% CI 0.82 to 0.92, p<0.001) and adjusted (adjusted time ratio (aTR) 0.90, 95% CI 0.85 to 0.95, p<0.001) models. An interaction analysis revealed a significant interaction between mood instability and diagnostic group (likelihood ratio (LR test, p<0.001). When stratified by diagnostic group, the fully adjusted Weibull AFT models demonstrated a similar pattern to the Kaplan-Meier curves, with the only significant association between mood instability and time to discharge being a reduction in the ADHD group (table 2).

**Table 2:** Adjusted and unadjusted TRs for time to discharge from CAMHS across diagnostic groups

	**ASD (n=**7444)		**ADHD (n=**7678)		ASD + ADHD (n = 6136)	
	TR(95% CI)	aTR (95% CI)	TR (95% CI)	aTR (95% CI)	TR (95% CI)	aTR (95% CI)
Mood instability	0.89* (0.80 to 0.98)	0.89 (0.80 to 1.00)	0.64† (0.58 to 0.70)	0.76† (0.69 to 0.84)	1.09 (0.98 to 1.20)	1.07 (0.97 to 1.18)
Age at index date	0.96† (0.95 to 0.97)	0.95† (0.94 to 0.96)	0.90† (0.89 to 0.91)	0.92† (0.91 to 0.93)	0.93† (0.92 to 0.94)	0.93† (0.92 to 0.94)
Ethnicity (reference white)						
Black	1.02 (0.93 to 1.12)	1.04 (0.94 to 1.15)	0.88* (0.80 to 0.96)	0.87* (0.80 to 0.94)	0.93 (0.83 to 1.03)	0.98 (0.89 to 1.08)
Asian	0.99 (0.84 to 1.16)	1.02 (0.86 to 1.20)	0.79* (0.64 to 0.97)	0.98 (0.81 to 1.18)	0.90 (0.73 to 1.11)	1.06 (0.87 to 1.28)
Mixed	1.15* (1.01 to 1.31)	1.10 (0.96 to 1.25)	1.07 (0.95 to 1.21)	1.04 (0.93 to 1.16)	0.99 (0.86 to 1.12)	1.01 (0.89 to 1.14)
Not stated	1.29† (1.15 to 1.45)	1.23† (1.09 to 1.38)	1.09 (0.98 to 1.23)	1.43† (1.28 to 1.59)	0.54† (0.46 to 0.63)	0.72† (0.63 to 0.84)
Other	1.48† (1.16 to 1.89)	1.38* (1.08 to 1.75)	1.14 (0.92 to 1.40)	1.24* (1.02 to 1.51)	0.94 (0.77 to 1.16)	1.13 (0.93 to 1.37)
Neighbourhood deprivation (reference least)						
Second	1.05 (0.94 to 1.16)	1.05 (0.95 to 1.17)	1.01 (0.91 to 1.13)	0.92 (0.83 to 1.02)	1.19* (1.06 to 1.33)	1.20† (1.08 to 1.34)
Third	1.08 (0.98 to 1.20)	1.10 (0.99 to 1.23)	1.09 (0.98 to 1.22)	0.99 (0.90 to 1.10)	1.12* (1.00 to 1.26)	1.15* (1.03 to 1.28)
Fourth (most)	0.98 (0.89 to 1.09)	1.01 (0.91 to 1.12)	1.03 (0.93 to 1.14)	0.90* (0.81 to 0.99)	1.24† (1.11 to 1.39)	1.19† (1.07 to 1.32)
Gender (reference male)						
Female	1.27† (1.17 to 1.36)	1.34† (1.24 to 1.45)	0.79† (0.73 to 0.85)	1.02 (0.95 to 1.10)	0.93 (0.85 to 1.02)	1.15† (1.05 to 1.25)
Other	1.69 (0.93 to 3.07)	2.09* (1.14 to 3.82)	0.78 (0.35 to 1.72)	1.03 (0.50 to 2.13)	1.15 (0.53 to 2.51)	1.22 (0.59 to 2.49)
Co-occurring diagnosis						
Psychosis	1.11 (0.93 to 1.31)	1.24* (1.03 to 1.48)	0.96 (0.81 to 1.15)	1.16 (0.98 to 1.37)	1.03 (0.86 to 1.24)	1.04 (0.88 to 1.23)
Eating disorder	0.92 (0.75 to 1.12)	0.94 (0.76 to 1.15)	0.66† (0.52 to 0.84)	1.00 (0.79 to 1.26)	0.88 (0.66 to 1.19)	0.96 (0.72 to 1.26)
OCD	1.39† (1.18 to 1.63)	1.40† (1.18 to 1.66)	0.93 (0.70 to 1.23)	1.20 (0.92 to 1.57)	1.30* (1.01 to 1.68)	1.24 (0.98 to 1.57)
Affective disorder	0.95 (0.82 to 1.09)	0.97 (0.84 to 1.13)	0.47† (0.40 to 0.55)	0.80* (0.69 to 0.93)	0.93 (0.77 to 1.12)	1.11 (0.93 to 1.33)
Phobia	0.75* (0.57 to 0.99)	0.78 (0.59 to 1.02)	0.57* (0.38 to 0.86)	1.03 (0.71 to 1.51)	1.14 (0.81 to 1.63)	1.16 (0.84 to 1.60)
Anxiety	0.94 (0.84 to 1.06)	0.96 (0.85 to 1.09)	0.78† (0.67 to 0.90)	1.09 (0.95 to 1.26)	1.16* (1.00 to 1.35)	1.23* (1.06 to 1.42)
ID	0.73† (0.66 to 0.81)	0.76† (0.68 to 0.85)	1.07 (0.91 to 1.26)	1.07 (0.92 to 1.24)	0.98 (0.87 to 1.10)	0.92 (0.83 to 1.03)
Conduct disorder	0.66† (0.54 to 0.80)	0.72† (0.59 to 0.88)	0.83* (0.73 to 0.94)	0.74† (0.66 to 0.83)	1.23* (1.07 to 1.41)	1.11 (0.97 to 1.26)
Emotional disorder	0.89 (0.77 to 1.01)	0.89 (0.77 to 1.02)	0.77† (0.68 to 0.86)	0.83† (0.74 to 0.93)	0.89 (0.77 to 1.04)	0.92 (0.80 to 1.06)
Tic disorder	0.96 (0.67 to 1.37)	0.92 (0.65 to 1.32)	1.04 (0.74 to 1.45)	0.93 (0.69 to 1.26)	1.07 (0.79 to 1.44)	0.95 (0.72 to 1.25)
Medication use						
Antipsychotic	1.06 (0.92 to 1.23)	1.06 (0.90 to 1.25)	0.74* (0.58 to 0.94)	0.91 (0.72 to 1.15)	1.25* (1.07 to 1.47)	1.07 (0.90 to 1.26)
Antidepressant	1.22† (1.08 to 1.38)	1.24* (1.08 to 1.43)	0.56† (0.46 to 0.68)	0.85 (0.69 to 1.05)	1.20* (1.02 to 1.42)	1.12 (0.94 to 1.34)
ADHD medication	N/A	N/A	2.25† (2.10 to 2.41)	2.26† (2.10 to 2.43)	2.49† (2.30 to 2.69)	2.37† (2.19 to 2.57)
Hypnotic	1.11 (0.94 to 1.32)	1.08 (0.90 to 1.30)	1.08 (0.88 to 1.32)	0.98 (0.81 to 1.19)	1.46† (1.25 to 1.71)	1.13 (0.97 to 1.32)

* P<0.05.

† P≤0.001.

ADHD, attention-deficit/hyperactivity disorder; ASD, autism spectrum disorder; aTR, adjusted time ratio; CAMHS, child and adolescent mental health services; ID, intellectual disability; N/A, not applicable; OCD, obsessive-compulsive disorder; TR, time ratio.

Sensitivity analyses stratified by ADHD medication status showed that, in the ADHD group, mood instability was associated with earlier discharge only among children not receiving medication (aTR 0.70, 95% CI 0.61 to 0.79, p<0.001), while there was no significant effect in those on medication (aTR 0.88, 95% CI 0.77 to 1.00, p>0.05). In the ASD+ADHD group, mood instability was not significantly linked to time to discharge in the subgroup not receiving ADHD medication (aTR 1.01, 95% CI 0.90 to 1.15, p>0.05), while it was associated with longer time to discharge in those receiving it (aTR 1.20, 95% CI 1.02 to 1.40, p<0.05).

### Annual CAMHS use

**Figure 1:**
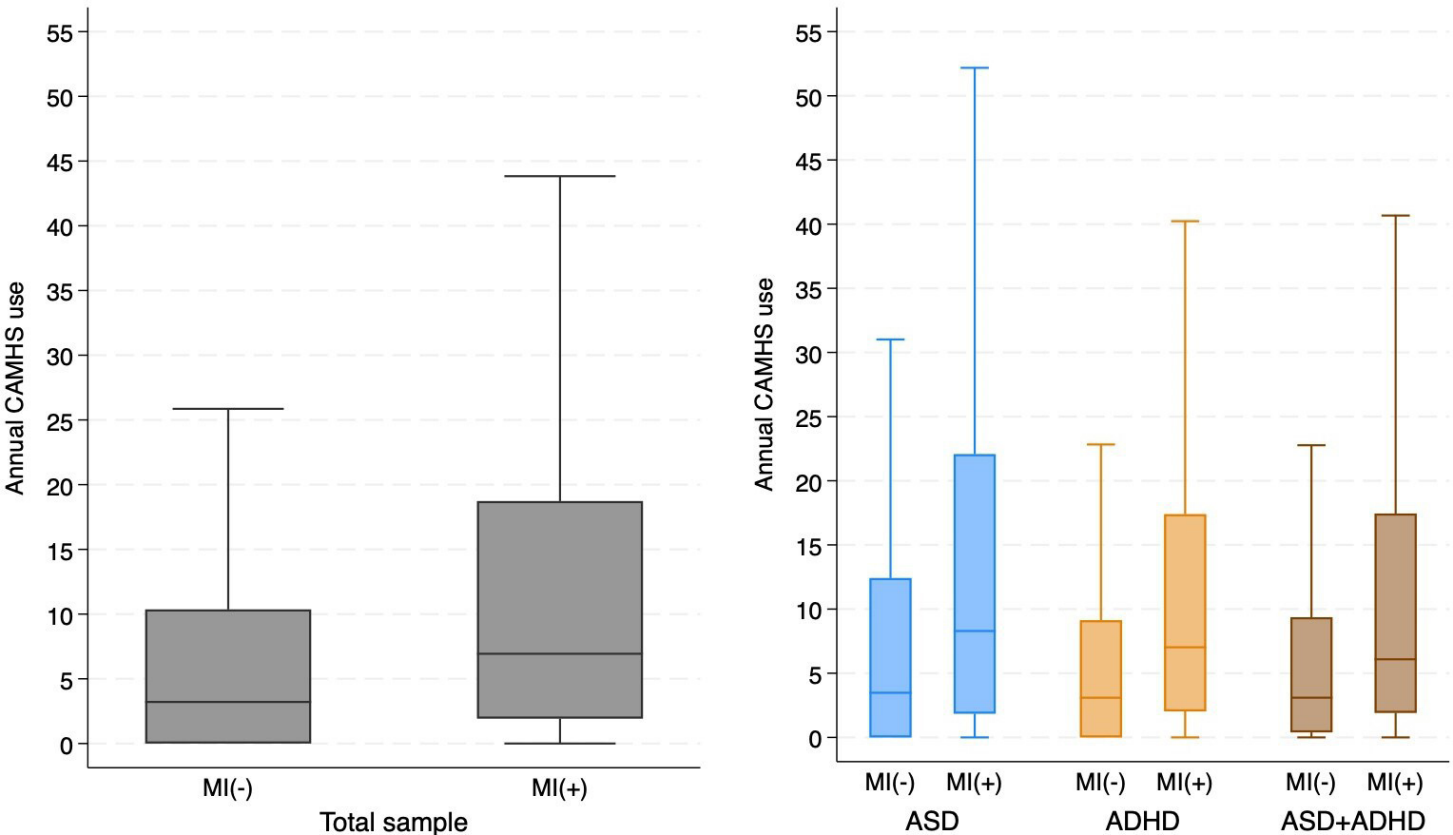
Median annual CAMHS use stratified by baseline MI. Given the high variance and skewness in the data, values were visualised in medians and IQRs to more accurately reflect typical service use. Horizontal lines within each box denote median values; boxes extend from the first to the third quartile of each group’s distribution of values; vertical extending lines represent the most extreme values within 1.5 IQR of each group. Median (IQR) values of annual CAMHS appointments were as follows: total sample: MI (–): 3.21 (0–10.35), MI (+): 6.93 (1.92–18.73); ASD: MI (–): 3.47 (0–12.41), MI (+): 8.29 (1.84–22.06); ADHD: MI (–): 3.09 (0–9.13), MI (+): 7.02 (2.02–17.39); ASD+ADHD: MI (–): 3.09 (0.39–9.36), MI (+): 6.08 (1.91–17.44). ADHD, attention-deficit/hyperactivity disorder; ASD, autism spectrum disorder; CAMHS, child and adolescent mental health services; MI, natural language processing-identified mood instability.

Figure 1 shows the mood instability-stratified median annual CAMHS use in the total sample and within each diagnostic group, presented as box plots. CYP with baseline mood instability had higher median annual CAMHS use in all groups (p<0.001).

Univariate negative binomial regression analyses showed that mood instability was associated with a significant increase in postdiagnostic annual CAMHS use in the whole sample (incidence rate ratio (IRR) 1.32, 95% CI 1.23 to 1.42, p<0.001), which persisted in the fully adjusted model (aIRR 1.32, 95% CI 1.23 to 1.42, p<0.001). An interaction analysis further indicated that mood instability significantly modified the effect of diagnostic group (ASD, ADHD and ASD+ADHD) on annual CAMHS use (LR test, p<0.001).

Table 3 presents the crude and adjusted IRRs for the association between mood instability and annual CAMHS use, stratified by diagnostic group. The mood instability-associated increase in annual CAMHS use was most pronounced among the ADHD group, followed by ASD+ADHD and ASD.

**Table 3:** Unadjusted and adjusted IRRs for annual CAMHS use across diagnostic groups

	**ASD (n=**7444)		**ADHD (n=**7678)		ASD+ **ADHD** (n= 6136)	
	IRR (95% CI)	aIRR (95% CI)	IRR (95% CI)	aIRR (95% CI)	IRR (95% CI)	aIRR (95% CI)
Mood instability	1.19* (1.05 to 1.36)	1.24† (1.08 to 1.42)	1.64† (1.44 to 1.85)	1.47† (1.30 to 1.67)	1.13* (1.00 to 1.28)	1.27† (1.12 to 1.44)
Age at index date	0.94† (0.93 to 0.95)	0.90† (0.89 to 0.91)	0.95† (0.93 to 0.96)	0.92† (0.91 to 0.93)	0.87† (0.86 to 0.89)	0.85† (0.83 to 0.86)
Gender (reference male)						
Female	1.28† (1.16 to 1.40)	1.19† (1.08 to 1.31)	1.20† (1.10 to 1.32)	1.25† (1.14 to 1.38)	0.91 (0.82 to 1.01)	1.14* (1.02 to 1.28)
Other	1.65 (0.87 to 3.13)	1.30 (0.69 to 2.44)	3.45† (1.44 to 8.23)	5.31† (2.32 to 12.14)	2.57*(1.19 to 5.52)	5.37† (2.39 to 12.04)
Ethnicity (reference white)						
Black	1.32† (1.17 to 1.49)	1.25† (1.11 to 1.42)	1.45† (1.29 to 1.62)	1.64† (1.47 to 1.83)	1.66† (1.44 to 1.91)	1.43† (1.26 to 1.64)
Asian	1.25* (1.01 to 1.54)	1.27* (1.02 to 1.57)	1.22 (0.95 to 1.57)	1.40* (1.10 to 1.79)	0.81 (0.64 to 1.03)	0.91 (0.72 to 1.14)
Mixed	1.21* (1.02 to 1.43)	1.11 (0.95 to 1.30)	1.20* (1.04 to 1.38)	1.23* (1.08 to 1.40)	0.74† (0.64 to 0.87)	0.76† (0.65 to 0.89)
Not stated	0.57† (0.49 to 0.67)	0.61† (0.52 to 0.71)	0.40† (0.35 to 0.46)	0.50† (0.43 to 0.57)	0.45† (0.36 to 0.57)	0.55† (0.44 to 0.69)
Other	1.08 (0.80 to 1.45)	1.23 (0.93 to 1.63)	0.60† (0.48 to 0.76)	0.68† (0.55 to 0.85)	0.86 (0.67 to 1.10)	0.90 (0.71 to 1.15)
Neighbourhood deprivation (reference least)						
Second	1.13 (.99 to 1.29)	1.06 (0.93 to 1.21)	0.76† (0.66 to 0.87)	0.71† (0.63 to 0.81)	0.86* (0.75 to 0.99)	0.81* (0.70 to 0.92)
Third	1.03 (0.91 to 1.18)	0.98 (0.85 to 1.11)	0.77† (0.67 to 0.88)	0.69† (0.61 to 0.79)	0.67† (0.58 to 0.77)	0.63† (0.55 to 0.73)
Fourth (most)	1.19* (1.04 to 1.36)	0.96 (0.84 to 1.10)	0.98 (0.86 to 1.12)	0.83* (0.73 to 0.94)	1.59† (1.37 to 1.84)	1.09 (0.94 to 1.26)
Co-occurring diagnosis						
Psychosis	1.00 (0.81 to 1.24)	0.84 (0.67 to 1.04)	1.77† (1.41 to 2.22)	1.23 (0.98 to 1.53)	1.54† (1.23 to 1.93)	1.50† (1.21 to 1.85)
Eating disorder	3.99† (3.06 to 5.21)	4.93† (3.79 to 6.42)	2.12† (1.59 to 2.81)	2.33† (1.78 to 3.06)	1.79† (1.30 to 2.47)	1.76† (1.28 to 2.42)
OCD	0.91 (0.75 to 1.10)	1.24* (1.02 to 1.52)	0.92 (0.67 to 1.25)	1.22 (0.90 to 1.64)	1.03 (0.79 to 1.35)	1.15 (0.88 to 1.51)
Affective disorder	2.70† (2.23 to 3.27)	3.09† (2.55 to 3.73)	2.40† (1.98 to 2.91)	2.96† (2.44 to 3.60)	2.77† (2.16 to 3.55)	2.96† (2.36 to 2.71)
Phobia	1.64* (1.15 to 2.34)	1.47* (1.03 to 2.09)	5.38† (3.10 to 9.34)	3.63† (2.16 to 6.08)	0.75 (0.50 to 1.13)	0.84 (0.56 to 1.26)
Anxiety	2.05† (1.76 to 2.39)	2.08† (1.79 to 2.42)	2.57† (2.13 to 3.09)	2.57† (2.15 to 3.08)	1.59† (1.35 to 1.88)	1.74† (1.47 to 2.07)
ID	1.37† (1.18 to 1.59)	1.49† (1.28 to 1.73)	1.20 (0.97 to 1.50)	1.13 (0.91 to 1.39)	1.49† (1.26 to 1.76)	1.12 (0.96 to 1.31)
Conduct disorder	1.89† (1.44 to 2.48)	2.08† (1.59 to 2.72)	2.40† (2.01 to 2.85)	2.38† (2.02 to 2.82)	0.72† (0.61 to 0.86)	0.74† (0.62 to 0.88)
Emotional disorder	2.17† (1.81 to 2.60)	2.22† (1.86 to 2.65)	2.33† (2.01 to 2.70)	2.27† (1.96 to 2.62)	1.72† (1.44 to 2.05)	1.69† (1.42 to 2.01)
Tic disorder	4.80† (2.92 to 7.87)	3.81† (2.32 to 6.23)	0.89 (0.59 to 1.34)	0.76 (0.51 to 1.12)	1.22 (0.83 to 1.78)	0.96 (0.66 to 1.39)
Medication use						
Antipsychotic	1.37* (1.11 to 1.69)	1.36* (1.09 to 1.71)	1.50* (1.13 to 2.00)	1.40* (1.02 to 1.93)	0.67† (0.55 to 0.81)	1.03 (0.83 to 1.27)
ADHD medication	N/A	N/A	1.70† (1.55 to 1.86)	1.80† (1.65 to 1.98)	1.40† (1.26 to 1.56)	1.45† (1.30 to 1.61)
Antidepressant	1.02 (0.88 to 1.19)	0.90 (0.75 to 1.07)	2.25† (1.79 to 2.83)	1.60† (1.25 to 2.04)	0.90 (0.74 to 1.08)	1.33* (1.08 to 1.64)
Hypnotic	1.23 (0.98 to 1.55)	1.35* (1.05 to 1.73)	1.76† (1.38 to 2.25)	1.59† (1.22 to 2.08)	0.62† (0.52 to 0.73)	0.68† (0.56 to 0.82)

* P<0.05,

† P≤0.001.

ADHD, attention-deficit/hyperactivity disorder; ASD, autism spectrum disorder; CAMHS, child and adolescent mental health services; ID, intellectual disability; IRR, incidence rate ratio; N/A, not applicable; OCD, obsessive-compulsive disorder.

In the ADHD group, sensitivity analyses revealed a greater increase in annual CAMHS use associated with mood instability among children not receiving ADHD medication (aIRR 1.67, 95% CI 1.47 to 1.89, p<0.001) than among those receiving medication (aIRR 1.24, 95% CI 1.10 to 1.39, p<0.001). In the ASD+ADHD group, mood instability was again linked to increased service use in both subgroups, with a stronger association observed in those who are not on ADHD medication (aIRR 1.60, 95% CI 1.40 to 1.82, p<0.001) than in those receiving it (aIRR 1.19, 95% CI 1.05 to 1.34, p<0.05).

## Discussion

Our study showed that mood instability at the time of index diagnosis was significantly associated with increased CAMHS use among CYP diagnosed with ASD and/or ADHD, even after adjusting for key sociodemographic and clinical factors including age, gender, ethnicity, socioeconomic status, co-occurring diagnoses and medication use. We also demonstrated the differential effects of mood instability across neurodevelopmental diagnostic profiles. While mood instability was associated with a 24% shorter time to discharge from CAMHS in the ADHD group, it was linked to a stark 47% increase in the annual CAMHS use for these CYP. On the other hand, mood instability was not associated with any significant change in the discharge timelines of autistic children regardless of their co-occurring ADHD status, while it was linked to an increase of 24% (ASD) and 27% (ASD+ADHD) in annual CAMHS use in these groups.

To our knowledge, this is the first study to date to examine mood instability specifically as a potential determinant of CAMHS use patterns in CYP with neurodevelopmental conditions; yet our findings align with previous research linking broader emotion dysregulation with increased mental health service utilisation in this population. For instance, Conner *et al* also reported that impaired emotion regulation was associated with higher psychiatric service use in autistic CYP,^54^ and Bierens *et al* found that emotion dysregulation predicted longer treatment duration in children with ASD.^20^ Emotional lability, a construct closely related to mood instability, was also linked to greater treatment use in children with ADHD.^19^ Furthermore, higher dysregulation scores in ADHD youth were associated with later adult mental health service utilisation.^55^ Importantly, previous studies typically assessed emotion dysregulation with psychometric tools, either using dedicated instruments like the Emotion Dysregulation Inventory or subscales of broader child mental health measures such as the Child Behaviour Checklist, Strengths and Difficulties Questionnaire or Conners Rating Scales.^19 20 54 55^ Due to the absence of emotion dysregulation measures implemented in routine clinical care, we instead used an NLP approach to identify mood instability as documented in clinical records. Despite these methodological differences, our findings converge with questionnaire-based evidence, supporting the potential of NLP as a scalable method to detect emotion dysregulation-related constructs within CAMHS EHRs. While the NLP-identified mood instability rates in our sample were lower than literature estimates of emotion dysregulation in ASD or ADHD,^16 17^ this is expected given the age range studied and because mood instability represents a discrete facet of the broader dysregulation spectrum.

The varying extent and nature of mood instability’s effect on CAMHS use patterns may partly stem from different clinical manifestations across ASD and ADHD. Mood instability was associated with the largest increase in CAMHS use in the ADHD group, where it also corresponded to a shorter time to discharge. In ADHD, compounded by the impulsivity inherent to the condition, mood instability may be expressed in a more externalising manner that activates healthcare input.^56^ As Ward and Curran showed in their study, emotion dysregulation in CYP with ADHD symptoms can lead to crisis presentations such as self-harm that signal high clinical risk,^57^ likely prompting intensive but short-lived CAMHS involvement. A recent UK consensus statement on ADHD also flagged emotion dysregulation as a trigger for CAMHS inclusion, which supports the pattern we identified in our cohort.^10^ In the ASD group, mood instability was associated with elevated annual CAMHS use without any significant impact on time to discharge. In autistic children, emotion dysregulation may present as a more enduring pattern that is intertwined with core features of autism, such as sensory sensitivities.^17 21^ It has also been linked to avoidant coping strategies, and even when expressed through meltdowns, these difficulties may often be perceived as intrinsic to autism, hindering access to targeted support.^32 58 59^ This dysregulation profile may explain the more moderate increase in mood instability-associated CAMHS use in autism. Children with ASD+ADHD showed an intermediate pattern, with mood instability-related CAMHS use increase falling between ASD and ADHD groups. Although this may appear counterintuitive, as dual diagnoses typically exacerbate healthcare demand,^60^ one possible explanation is that these CYP actually represent a high complexity group with additional difficulties accessing support due to compounded challenges. Similarly, the reduction in the ADHD group or lack of extension in time to discharge in autistic CYP despite elevated CAMHS use may indicate that mood instability undermines sustained service involvement. While no previous work has specifically investigated the impact of mood instability on CAMHS discharge timelines, our results support the emerging evidence suggesting that baseline emotion dysregulation can predict higher dropout rates from community mental healthcare in other conditions where both ASD and ADHD are common co-occurrences.^61^ Recent meta-analytic evidence also supports this, linking reduced community CAMHS engagement with multiple problems.^62^

Beyond differences in clinical presentation, how mood instability translates into service involvement is also likely shaped by the configuration of neurodevelopmental CAMHS in the UK and what support is on offer for these CYP. Following an ADHD diagnosis, the mainstay of CAMHS provision centres on medication, typically managed through medication review clinics run by medical or non-medical prescribers.^63 64^ When ADHD co-occurs with autism, the CYP can access the ASD pathway where multidisciplinary CAMHS input is more readily available.^65^ As explained earlier, SLaM CAMHS follows a similar overall service organisation. Data on the content of individual contacts or on specific interventions for emotion dysregulation were not available to us. However, pathway-level documents indicate that specialist and ASD-informed support for autism-related mental health presentations is offered within the ASD pathway, including an intensive intervention framework for autistic young people with severe emotion dysregulation, whereas no comparable provision is described in the ADHD pathway. These differences in support availability may help explain the CAMHS use patterns when stratified by ADHD medication. In the ADHD group, mood instability was associated with the steepest increase in CAMHS use (67%) but a discharge timeline reduced by 30% among those not receiving ADHD medication, suggesting short-term, crisis-driven bursts of CAMHS input. In the ASD+ADHD subgroup not receiving ADHD medication, mood instability was associated with a 60% increase in CAMHS use, but this time also linked with a 20% significant extension in time to discharge. These patterns imply a more stabilised care trajectory in CYP with ADHD (with or without co-occurring ASD) if they are receiving ADHD medication, which is consistent with prior evidence.^66^ National guidelines recommend at least annual specialist reviews for children prescribed ADHD medication, leading to more sustained CAMHS use in this group.^35^ Considering ADHD medication has been shown to improve emotion dysregulation, the attenuated increase in mood instability-related CAMHS use among CYP accessing ADHD pharmacotherapy aligns with clinical expectations.^67^ These findings also suggest that when evidence-based interventions for the underlying condition, such as medication for ADHD, are integrated into the care plan, mood instability may place less additional burden on CAMHS. While we cannot ascertain the reasons for not being on medication, contraindications and poor family support are among the main considerations when initiating a medication and may indicate additional complexity.^35 68^ Notwithstanding, differences in mood instability-related time-to-discharge patterns between medicated ADHD and ADHD+ASD groups suggest that the availability of non-pharmacological emotion regulation support is highly variable, particularly for those who cannot access ASD pathways, where multidisciplinary provision for co-occurring difficulties is more embedded.^69^ Taken together, our findings suggest that within the existing care pathways CYP in the ADHD group who do not receive medication may be at a structural disadvantage, where added transdiagnostic complexity such as mood instability is associated with earlier service disengagement.

### Strengths and limitations

One of the main strengths of this study was its substantial sample size of 21 906 children and adolescents with ASD and/or ADHD, making this, to our knowledge, the largest clinical study to date to examine associations between emotion dysregulation, specifically mood instability, and mental health service utilisation in this group. Using routinely collected clinical data enabled a real-world study design without research criteria filtering, while also providing rich individual and contextual information to improve model accuracy. Although we could not account for changes in service provision or policy over the observation period, our pragmatic design allowed us to test whether baseline mood instability predicted future CAMHS use in children with neurodevelopmental conditions. Using NLP analysis instead of collecting questionnaire-based responses from participants helped minimise response and attrition biases, which often limit the generalisability of naturalistic studies reliant on psychometric measure completion.^70^ While diagnostic ascertainment is a common challenge in EHR-based research, NLP methods were also instrumental in substantially improving the availability of diagnostic data in this study.

This research also has limitations that should be considered when interpreting the findings. EHR analysis is inherently constrained by the extent of clinical documentation—poor clinical documentation is a recognised limitation of NLP-based clinical research.^38^ In addition, differences in symptom capture may also have arisen from variation in assessment practices. Although the NLP model we employed was effectively used in adolescent and young adult populations in previous work, it was mainly developed in EHRs of adults with severe mental illness.^22 26^ As a result, we may have underdetected mood instability in the sample, either due to missing information in clinical records or differences in terminology between CAMHS and adult mental health documentation. While the F1-score of this NLP algorithm is reassuring regarding overall performance, its relatively lower recall may also have contributed to underdetection. Nevertheless, this may mean that the true associations between mood instability and CAMHS use could be stronger than estimated, as misclassification of dichotomous exposures often biases associations towards the null.^71^ While coding mood instability as a binary variable has introduced its own limitations (eg, not taking into account nuances in presentation such as severity), this approach was used in previous CRIS work specifically to reduce sensitivity to differences in documentation volume and frequency of mentions.^22 72^ Importantly, the pattern of our results is not consistent with a simple documentation-volume artefact. If volume of documentation (which is directly related to number of contacts) were the primary driver, we would expect the strongest associations between mood instability and CAMHS use in the ASD+ADHD group, where annual CAMHS use is highest (table 1). Instead, we observed the largest associations in the ADHD-only group, which has lower CAMHS use. The differential pattern of significance and effect sizes we found for our outcomes across diagnostic groups suggests that the findings are unlikely to be spurious or solely attributable to documentation volume. Nevertheless, confounding by document-volume remains a common limitation and concern in NLP-based health record analyses. Where possible, future work could also use NLP tools that identify clinically ‘inert’ concepts as negative control exposures to further test robustness against documentation-related confounding—this approach was not feasible in this study as such applications are not currently available within CRIS. We lacked exact timing or repeated measures of mood instability to account for persisting problems, or data regarding psychosocial interventions, both of which may have influenced CAMHS use patterns. Overall, more standardised documentation of the clinical appointment content would enable more nuanced analyses of service use patterns. The number of contacts tends not to be evenly distributed over time, which may have skewed contact rates towards those with shorter follow-up. For this study, face-to-face contact was selected as our service-use marker as these were the most consistently and reliably recorded across the entire study period, and to avoid misclassification arising from telephone or other remote contacts, which are also commonly used for administrative purposes. As EHR systems evolve to more fully capture the mode and purpose of appointments in structured fields, temporally stratified analyses of contact type will become more feasible. Such analyses will be particularly informative once sufficiently large cohorts have accumulated to support robust stratified comparisons over time. The absence of discharge reason codes limited our ability to interpret time-to-discharge outcomes, as we could not firmly distinguish between premature service disengagement and discharge as a result of treatment success. These limitations could be mitigated by tailoring NLP approaches for CAMHS patients, augmented by standardised psychometric assessments to capture severity and context, and by improving recording practices to include reason for discharge for each child.

### Clinical implications and future research

This study found that presenting with mood instability, a key trait of emotion dysregulation, at the time of diagnosis is significantly associated with increased CAMHS use among CYP with ASD and/or ADHD. This effect is seen across diagnoses with some variation in time-to-discharge patterns, which may stem from differences in symptom presentations and care pathway configuration. While we did not conduct a causal inference study, these findings suggest important clinical implications for CAMHS-accessing CYP with ASD and/or ADHD, given the size and granularity of the dataset. We argue that emotion dysregulation assessment should be integral to neurodevelopmental assessments to inform care planning. Evidence-based interventions targeting core difficulties of underlying neurodevelopmental conditions, such as medication in ADHD, may mitigate CAMHS needs related to emotion dysregulation. Finally, care pathway differences for ADHD and ASD require further consideration when additional complexity, such as emotion dysregulation, is present, to ensure appropriate support and adequately sustained service engagement. Our findings reinforce previous evidence from psychometric research and support the potential of NLP-based methods for identification of emotion dysregulation-related constructs in clinical records. Future work should aim to refine such scalable tools to better capture key determinants of increased mental health needs, within routine clinical practice, to ultimately optimise care in neurodevelopmental CAMHS.

## Supplementary material

10.1136/bmjment-2026-302488 online supplemental file 1

## Data Availability

Data may be obtained from a third party and are not publicly available.
