# Supplementary material for: Mood instability and mental health service use in autism and attention-deficit/hyperactivity disorder: a natural language processing analysis of CRIS electronic healthcare records from 21 906 children and adolescents
Source: BMJ Ment Health. 2026 Jun 18;29(1):e302488. doi: 10.1136/bmjment-2026-302488 (PMC13288977; doi:10.1136/bmjment-2026-302488)
Supplement: online supplemental file 1 [file bmjment-29-1-s001.pdf]

Table of Contents

FIGURE S1. STUDY SAMPLE DERIVATION FROM TOTAL SLAM CAMHS COHORT 2

TABLE S1. MISSING VALUES 2

SEARCH TERMS FOR MOOD INSTABILITY NATURAL LANGUAGE PROCESSING APPLICATION: 3

PRECISION, RECALL AND F1-SCORE FORMULAS: 3

PSYCHIATRIC DIAGNOSES AND CORRESPONDING ICD-10 CODES: 3

FIGURE S2. KAPLAN-MEIER CURVES FOR TIME-TO-DISCHARGE, STRATIFIED BY BASELINE MOOD INSTABILITY 4

TABLE S2. DIAGNOSTIC GROUP - MOOD INSTABILITY INTERACTIONS FOR SERVICE USE OUTCOME 4

TABLE S3. DIAGNOSTIC GROUP - MOOD INSTABILITY INTERACTIONS FOR TIME-TO-DISCHARGE OUTCOME 5

TABLE S4. MODEL FIT INDICES FOR ALTERNATIVE PARAMETRIC SURVIVAL DISTRIBUTIONS 5

SLAM CAMHS CARE PATHWAYS: 5

Figure S1. Study sample derivation from total SLaM CAMHS cohort

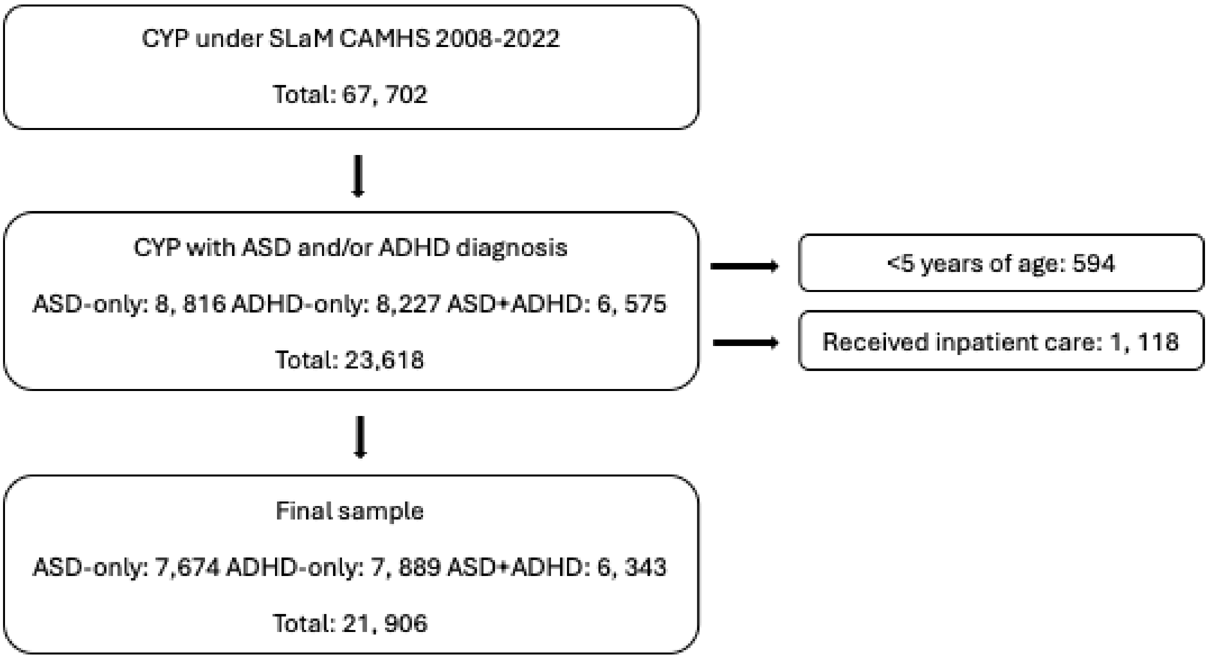

Table S1. Missing values

	Total	ASD	ADHD	ASD+ADHD
**Gender**	24	11	5	8
**Ethnicity**	27	6	15	6
**Neighbourhood Deprivation**	596	214	190	192

Search terms for Mood Instability Natural Language Processing application:

Chang* [0-2 words in between] *mood*
Extremes [0-2 words in between] *mood*
Fluctuat* [0-2 words in between] *mood*
Instability [0-2 words in between] *mood*
labil [0-2 words in between] mood
Rapid cycling [0-2 words in between] mood
swings [0-2 words in between] mood
unpredictable [0-2 words in between] mood
unsettled [0-2 words in between] mood
unstable [0-2 words in between] mood
variable [0-2 words in between] mood
variation [0-2 words in between] mood
volatile [0-2 words in between] mood
mood [0-2 words in between] chang*
mood [0-2 words in between] Extremes
mood [0-2 words in between] fluctuat*
mood [0-2 words in between] Instability
mood [0-2 words in between] *labil*
mood [0-2 words in between] Rapid cycling
mood [0-2 words in between] *swings*
mood [0-2 words in between] *unpredictable*
mood [0-2 words in between] Unsettled
mood [0-2 words in between] Unstable
mood [0-2 words in between] *variable*

Precision, recall and F1-score formulas:

Precision = True Positives /(True Positives + False Positives)
Recall = True Positives /(True Positives + False Negatives)
F1-score= 2xPrecisionXRecall/(Precision + Recall)

Psychiatric diagnoses and corresponding ICD-10 codes:

Autism Spectrum Disorder (ICD-10 F84.0-F84.9)
Psychosis (ICD-10 F1x.5, F20–F29, F31, F32.3, F33.3)
Eating Disorder (ICD-10 F50)
Obsessive-Compulsive Disorder (ICD-10 F42)
Phobia (ICD-10 F40.0-F40.9)
Anxiety (ICD-10 F41.0-F41.9)
Intellectual Disability (ICD-10 F70.0-F70.9, F71.0-F71.9, F72.0-F72.9, F73.0-F73.9, F78.0-F78.9, F79.0-F79.9)
Conduct Disorder (ICD-10 F91.0-F91.9, F92.0-F92.9)
Emotional Disorder (ICD-10 F93.0-F93.9)
Tic Disorders (ICD-10 F95.0-F95.9)

Figure S2. Kaplan-Meier curves for time-to-discharge, stratified by baseline mood instability

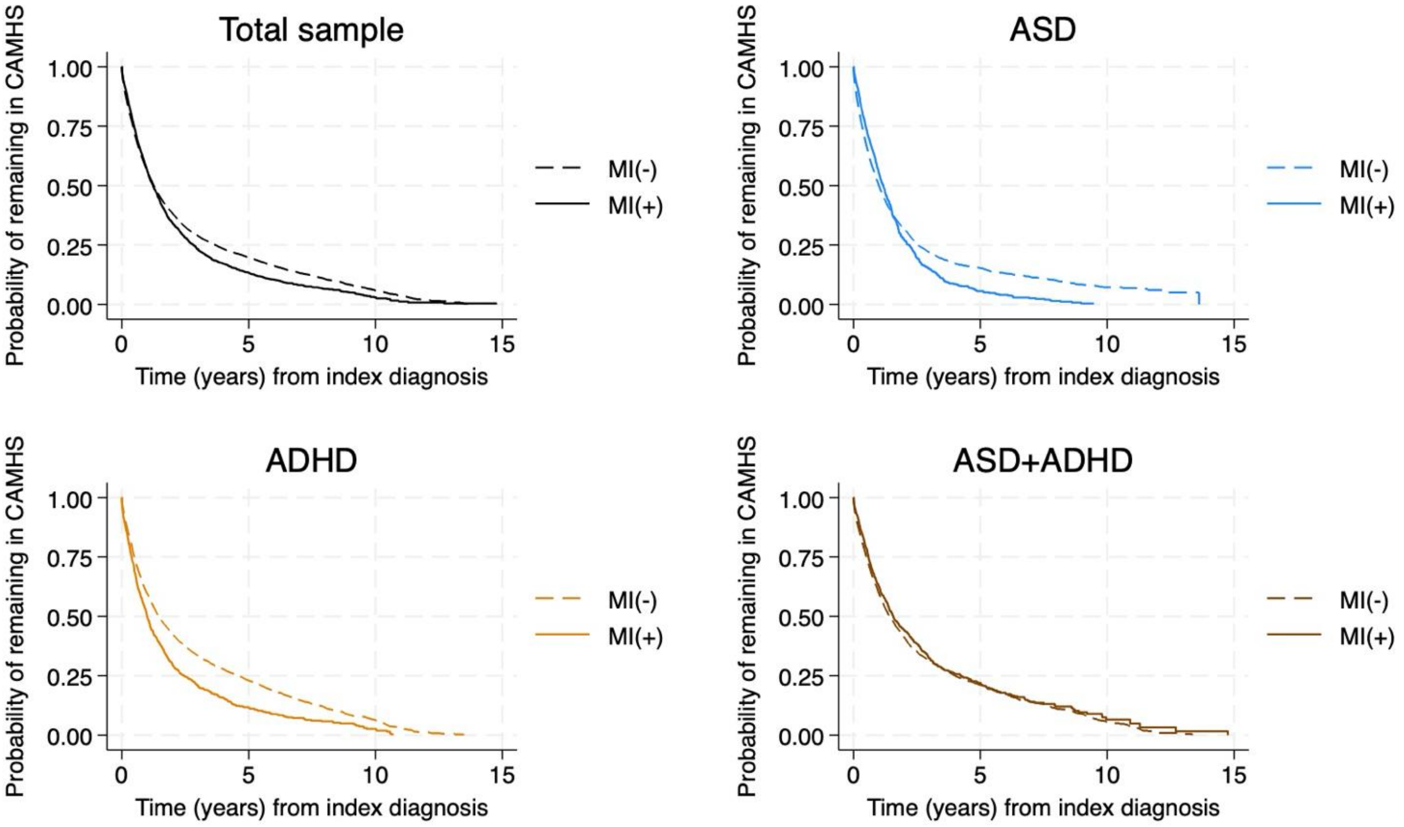

ASD: Autism Spectrum Disorder, ADHD: Attention Hyperactivity Disorder, MI: NLP-identified mood instability

Table S2. Diagnostic Group - Mood Instability interactions for Service Use outcome

	**IRR [95% CI]**	**aIRR [95% CI]**
Mood instability	1.63**[1.43-1.85]	1.75**[1.54-1.99]
ASD-only (reference ADHD)	1.29**[1.20-1.38]	1.54**[1.44-1.66]
ASD+ADHD (reference ADHD)	1.45**[1.34-1.56]	1.36**[1.26-1.47]
ASD-only#Mood instability	.74**[.62-.88]	.71**[.59-.84]
ASD+ADHD#Mood instability	.69**[.58-.82]	.61**[.51-.73]

*ASD: Autism Spectrum Disorder; ADHD: Attention Deficit Hyperactivity Disorder; IRR: Incidence Rate Ratio; CI: Confidence Interval; *p<0.05, **p ≤0.001*

Table S3. Diagnostic Group - Mood Instability interactions for Time-to-Discharge outcome

	TR [95% CI]	aTR [95% CI]
ASD-only (reference ADHD)	.68**[.64-.71]	.99[.93-1.05]
ASD+ADHD (reference ADHD)	.95[.89-1.00]	.93*[.88-.98
Mood instability	.64**[.58-.70]	.68**[.62-.75]
ASD-only#Mood instability	1.38**[1.20-1.59]	1.40**[1.22-1.61]
ASD+ADHD#Mood instability	1.70**[1.47-1.96]	1.59**[1.39-1.82]

*ASD: Autism Spectrum Disorder; ADHD: Attention Deficit Hyperactivity Disorder; TR: Time Ratio; CI: Confidence Interval; *p<0.05, **p ≤0.001*

Table S4. Model fit indices for alternative parametric survival distributions

Distribution	df	AIC	BIC
Lognormal	30	67609.8	67848.74
Weibull	30	65796.59	66035.53
Exponential	29	67106.61	67337.58
Loglogistic	30	66472.72	66711.65

df: Degrees of freedom; AIC: Akaike Information Criterion; BIC: Bayesian Information Criterion

SLaM CAMHS Care Pathways:

1- Anxiety pathway
2- Autism Spectrum Disorder pathway
3- Psychosis pathway
4- Cognitive and neuropsychological assessment pathway
5- Mood pathway
6- Obsessive Compulsive Disorder pathway
7- Post-Traumatic Stress Disorder pathway
8- Self-harm pathway
9- Attention Deficit Hyperactivity Disorder pathway
10-Parenting pathway
